# Development and Validation of a Dissolution Test for Meloxicam and Pridinol Mesylate from Combined Tablet Formulation

**DOI:** 10.4103/0250-474X.65033

**Published:** 2010

**Authors:** S. E. Vignaduzzo, P. M. Castellano, T. S. Kaufman

**Affiliations:** Institute of Chemistry, Rosario (IQUIR, CONICET-UNR), Suipach 513, 52002LRK, Rosario, Argentine

**Keywords:** Dissolution test, meloxicam, method validation, pridinol mesylate, quality control of tablets

## Abstract

The association of meloxicam and pridinol is indicated for treating muscular contractures and low back pain. A dissolution test for the meloxicam-pridinol combined tablet formulation was developed and validated, using a suitable HPLC method for simultaneously quantitating both dissolved drugs. The optimized conditions include the use of USP apparatus 2 at a paddle rotation rate of 75 rpm and 900 ml of 50 mM phosphate buffer (pH= 7.5) as dissolution medium, at 37.0±0.5°. The test, which demonstrated to be robust against small changes in bath temperature, paddle rotation speed and pH of the dissolution medium, was applied to two different brands of tablets; the corresponding dissolution profiles were constructed and both brands showed to dissolve at least 75% of the drugs at the 45 min time point.

The dissolution test is a simple and useful *in vitro* tool that can provide valuable information about drug release similarity among different batches (manufacturing reproducibility) and brands (product performance similarity) of a product, and clues about the biological availability of a drug from its formulation; therefore, it is considered as one of the most important quality controls of solid pharmaceutical dosage forms[1].

Meloxicam (MEL) is a non-steroidal antiinflammatory drug, which is known to preferentially inhibit the enzyme cyclooxygenase-2 (COX-2) over COX-1[2]. The drug (fig. 1) is slowly but almost completely absorbed after oral administration[3,4]; according to the biopharmaceutics classification system[5], it can be included among the Class II compounds (low solubility and high permeability). Because of its effectiveness and good overall safety profile[6], MEL is prescribed for treating various arthritic and inflammatory conditions, including rheumatoid arthritis, osteoarthritis, ankylosing spondylitis and other joint diseases[7]. Pridinol mesylate (PRI) is a central anticholinergic drug[8], employed as a myotonolytic and spasmolytic agent in antistress therapy[9]. The drug is sparingly soluble in water. The pharmaceutical association of MEL and PRI is indicated for treating muscular contractures and low back pain[8,10].

**Fig. 1:**
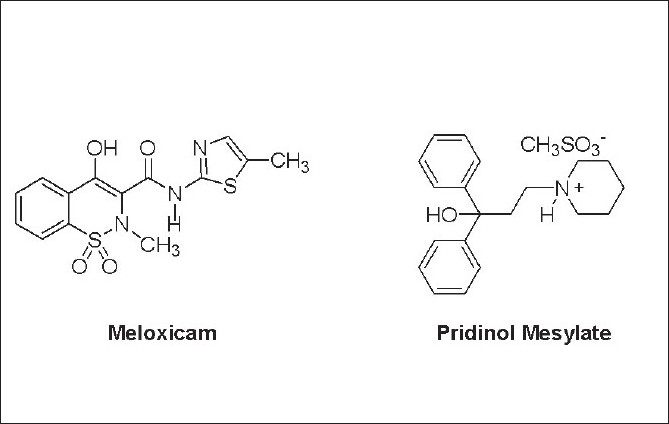
Chemical structures of meloxicam and pridinol mesylate

Dissolution testing of formulations containing poorly soluble drugs has experienced increasing interest in recent years, driven by the need of finding proper conditions for their routine quality control[11]. In addition, development and validation of dissolution tests for drugs present in low concentrations have also received special attention[12].

The solubility characteristics of MEL have been shown to result in poor dissolution, as well as in variations of its bioavailability[13], and have been linked to diminished tolerability and some gastric irritation upon oral administration[14]. Due to the importance of the problem, several solubilization and dissolution studies have been reported for this drug[15–18]. Meloxicam is official in the British Pharmacopoeia[19]; on the contrary, PRI has not been included yet in the most consulted Pharmacopoeias. A literature search revealed the lack of a validated dissolution test for the combined MEL-PRI formulation, and evidenced that published examples of validated dissolution tests for pharmaceutical associations are scarce[20–22]. Therefore, herein we report the development and validation of a dissolution test for tablets of the MEL-PRI association.

## MATERIALS AND METHODS

All experiments were performed with pharmaceutical-grade MEL and PRI (Saporiti, Buenos Aires, Argentina), analytical-grade reagents and HPLC-grade solvents. Buffer solutions were prepared with double distilled water. All dilutions were performed in standard volumetric flasks. Solvents and solutions were filtered through 0.45 mm nylon filters before use. The pharmaceutical preparations, declaring to contain 15 mg MEL and 4 mg PRI, were obtained from local drugstores. Optimization experiments were carried out with Brand 1.

The dissolution experiments were performed in a Hanson SR8-Plus dissolution Test Station, configured as USP apparatus 2 (paddles). The dissolution samples were analyzed as previously described[23], employing a Varian ProStar HPLC system which consisted of two pumps, a manual injector fitted with a 20 µl loop, a 250×4.6 mm C18 column (Luna, Phenomenex, 5 µm particle size) and a Varian Prostar 325 variable dual-wavelength UV/Vis detector, set at 225 nm. The mobile phase was a 51:9:40 (v/v/v) mixture of methanol, isopropanol and 50 mM potassium phosphate buffer (pH 5.9), pumped at a flow rate of 1.0 ml/min. Data were acquired and processed employing Varian Star v. 6.0 software.

### Standard and working stock solutions of MEL and PRI:

The stock solution of standard MEL (702 mg/l) was prepared in a 50 ml volumetric flask by dissolving an accurately weighed amount of MEL in a mixture of methanol (20 ml) and 0.1 N NaOH (5 ml). The solution was completed to the mark with methanol. The stock solution of standard PRI (400 mg/l) was prepared in a 50 ml volumetric flask by dissolving an accurately weighed amount of the drug (20.0 mg) in methanol (25 ml) and completing to the mark with the same solvent. The working solutions of MEL and PRI were prepared in mobile phase by dilution of the corresponding standard stock solutions, to yield the analytes at final concentrations of 84.2 mg/l and 24.0 mg/l, respectively. Solutions for analyses containing mixtures of the analytes were prepared in 10 ml volumetric flasks, immediately before use, by appropriate dilution of the working solutions with mobile phase.

### Optimization and validation of the dissolution test:

Optimization was carried out employing 900 ml of 50 mM phosphate buffer as dissolution medium per vessel, thermostatized at 37.0±0.5°. Six tablets were processed in each dissolution experiment. The effect of the pH of the dissolution medium was studied at different levels (5.5, 6.5, 7.5 and 8.0). The effect of the paddle rotation speed was examined at 50, 60 and 75 rpm. Sink conditions were verified through the analysis of dissolution samples taken from a vessel, where amounts of the drug equivalent to three times those in the tablet (45 mg) were added.

Precision was studied at the instrumental (HPLC) precision, method repetability and intermediate precision levels. Instrumental precision was determined as the relative standard deviations (RSD) of the drug recoveries of triplicate injections of combined standard samples of the analytes at 50, 90 and 130 % concentration levels. Overall method repeatability, including tablet manufacturing variability, was determined as the RSD of the amounts of dissolved drugs from six tablets after 10, 30 and 45 min of dissolution time. Intermediate precision of the dissolution test was demonstrated by analysis of two sets of six tablets each, from the same lot in two different days, with independently prepared standard solutions, dissolution media and mobile phases. The robustness of the test was examined against small but deliberate variations of critical parameters, including bath temperature (35.0-39.0 °), pH of the dissolution medium (7.3-7.7) and paddle rotation speed (71-79 rpm).

### Dissolution studies; sample preparation and optimized dissolution test procedure:

One tablet was placed in each of the six vessels of the dissolutor, filled with 900 ml of 50 mM phosphate buffer (pH= 7.5), preheated at 37.0±0.5°, and the dissolution medium was stirred at 75 rpm. Aliquots of the dissolution medium (5 ml) were withdrawn at 5, 10, 20, 30, 45, and 60 min and filtered, discarding the first portions of the filtrates; 2 ml of the filtrates were transferred to 5 ml volumetric flasks and completed to the mark with methanol. The amounts of the dissolved drugs were determined by HPLC[23].

### Solution stability studies:

After completing the dissolution procedure, the solutions were stirred slightly at 37±0.5° for 3 h (24 h for the experiment at pH= 7.5). Aliquots of each dissolution medium were withdrawn at the initial time and then regularly up to 3 or 24 h. Samples, injected in triplicate, were evaluated against a freshly prepared standard solution of the analytes.

## RESULTS AND DISCUSSION

Factorial designs are known to provide good amounts of information with a reduced set of experiments; therefore, they are usually preferred to the more time-consuming batch to batch optimization strategies. However, owing that dissolution tests should be performed in runs of at least 6 units, in order to take into account variations in the manufacture of the dosage form, and since the dissolution station architecture prevents bath temperature and agitation speed to be set independently for each vessel, the dissolution stage of the test was optimized and validated employing a step-wise strategy.

In order to optimize the dissolution test, initial conditions were set in agreement with currently accepted practices[24,25]. The paddles setup was selected due to its inherent advantages over the basket system[26]. Selection of a proper dissolution medium was carried out were taken into account drug solubilities. Meloxicam is practically insoluble in water[19] and at pH values lower than 5.5, its solubility decreases sharply (2.1 mg/l at pH= 5 and 0.5 mg/l at pH= 4), hindering the attainment of sink conditions and the test itself[14]. This is because under these conditions MEL is a zwitterion (pKa= 1.09 and 4.18)[14], which has a large intramolecular multipole moment[27], due to the presence of oppositely charged groups within the molecule. On the other hand, dissolution media with pH higher than 8.0 are not recommended.

**Fig. 2:**
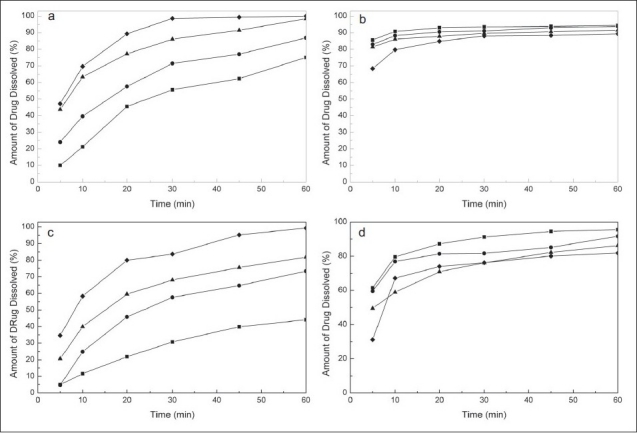
Effect of the pH of the dissolution medium. Dissolution profi les of MEL (meloxicam, a and c) and PRI (pridinol, b and d) in their combined tablet formulation at pH= 5.5 (—■—), pH= 6.5 (—●—), pH= 7.5 (—▲—), pH= 8.0 (—♦—). Bath temperature: 37.0±0.5°, volume of dissolution medium: 900 ml. Paddle rotation speed: 75 rpm (top) and 50 rpm (bottom).

The experimental results revealed that, regardless of the paddle rotation speed, the dissolution rate of MEL increased with increasing the pH of the medium (fig. 2). However, it was observed that the generic dissolution specification for conventional-release oral dosage forms of the BP ('no less than 75 % of the labeled amount in 45 min')[19], was met only at pH values of 7.5 and higher, and more confidently at 75 rpm (fig. 2a).

On the other side, PRI evidenced a smaller but opposite trend, showing a slight increase in the amounts being dissolved with the increasing acidity of the dissolution medium (fig. 2b). The improved dissolution of PRI at lower pH is probably due to the protonation of its piperidinic nitrogen (pKa ≈ 9.7). However, since dissolution rates of PRI higher than 75% at 45 min were attained under all test conditions, it was concluded that dissolution of MEL conditioned selection of the medium and that a pH value of 7.5 was the most suitable for the proposed method.

**Fig. 3:**
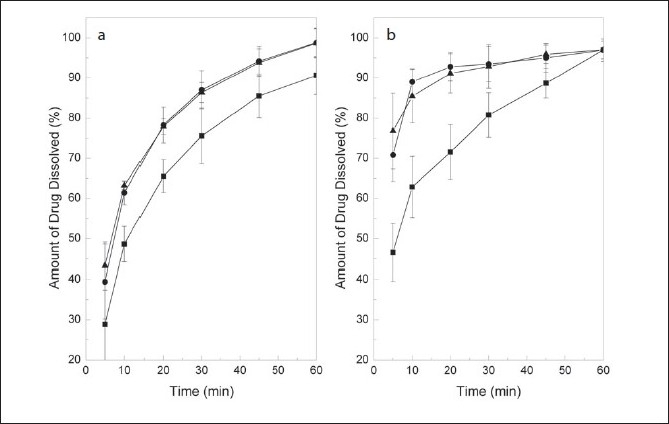
Effect of the paddle rotation speed. Dissolution profi les of MEL (meloxicam, a) and PRI (pridinol, b) in their combined tablet formulation at 50 rpm (—■—), 60 rpm (—●—) and 75 rpm (—▲—). Bath temperature: 37.0±0.5°, volume of dissolution medium: 900 ml, pH= 7.5

When the effect of paddle rotation rate was examined at pH 7.5, dissolution differences between 50 and 60 rpm were noticeable for both drugs (fig. 3), with increasing rotation speeds favouring dissolution. Interestingly, drug release rates were found statistically similar at 60 and 75 rpm, when a *t*-test comparison of the means was performed at each time point. However, it was concluded that 75 rpm was the best suited since it provided more consistent results. This rotation speed is usually employed for testing drug combinations[24].

The suitability of the dissolution stage of the test was demonstrated employing a valid HPLC method for the simultaneous determination of MEL and PRI[23]. The small pH differences between the dissolution media and the mobile phase demonstrated to have no effect on peak shapes, retention times and area under the curves of the analytes. Meloxicam, the least soluble of both drugs, exhibits a water solubility of 7.9 mg/l in its micronized form[15]; at pH= 8, MEL dissolves up to 1550 mg/l[14]. At the optimized dissolution pH the drug attained sink conditions[28,29].

In addition, samples' stability was evaluated in order to guarantee their chemical integrity during the analysis time. Deviations greater than ±2% from the initial analysis were not observed, and both analytes were stable for at least 3 h at pH= 5.5, 6.5 and 8.0, and at least 24 h at pH= 7.5. Furthermore, no degradation products were detected, confirming the stability of the samples.

Method precision was considered satisfactory, as stemmed from the results shown in Table 1. The absolute deviations among corresponding mean amounts of the dissolved drugs did not exceed 10% at time points below 85% of dissolution and were less than 5% for time points above this level, fully complying with typical requirements[24].

**TABLE 1:** PRECISION OF THE DISSOLUTION TEST

Parameters	Meloxicam			Pridinol		
Instrumental precision						
Drug concentration level (%)	50	90	130	50	90	130
RSD (%)	1.03	0.28	0.40	1.28	1.11	0.40
Overall method repeatability						
Mean amount of drug dissolved (%)^a^	61.4	83.0	99.7	76.3	83.2	96.0
RSD (%)	3.3	5.0	4.2	3.8	2.0	1.1
Intermediate precision						
Mean amount of drug dissolved (%)	24	45	88	50	73	91
Difference (%)	9.0	4.0	2.9	6.9	3.6	3.0

**Fig. 4:**
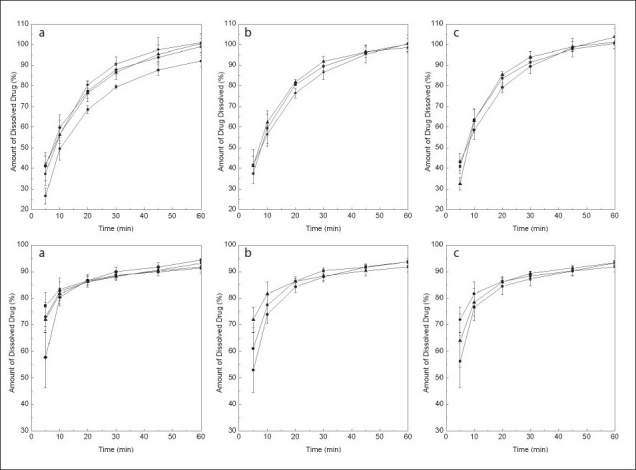
Robustness of the dissolution test. Dissolution profi les of MEL (meloxicam, top) and PRI (pridinol, bottom) against: a) bath temperature 35 ° (—■—), 36 ° (—●—), 37 ° (—▲—), 39 ° (—♦—); b) pH 7.3 (—■—), 7.5 (—●—), 7.7 (—▲—) and c) paddle rotation speed 71 rpm (—■—), 75 rpm (—●—), 79 rpm (—▲—)

When the robustness of the method was examined, not unexpectedly, higher data dispersion was observed at the initial times (fig. 4); however, these achieved average RSD values of 4.5 and 2.7% for MEL and PRI, respectively, at the 45 min time point, being considered satisfactory and complying with general official specifications (< 10%)[24].

Except for the dissolution of MEL at 35° (fig. 4a), which was noticeably lower than at slightly higher temperatures, probably due to a marked temperature-dependent solubility of the drug, the results were considered satisfactory. No significant differences were found among the amounts of dissolved drugs at the pre-specified time points under the different temperatures, with respect to those corresponding to the optimal experimental conditions, confirming the robustness of the test at a ±1° level. In addition, the optimized conditions proved to be robust with regards to pH of the dissolution medium and paddle rotation speed.

**Fig. 5:**
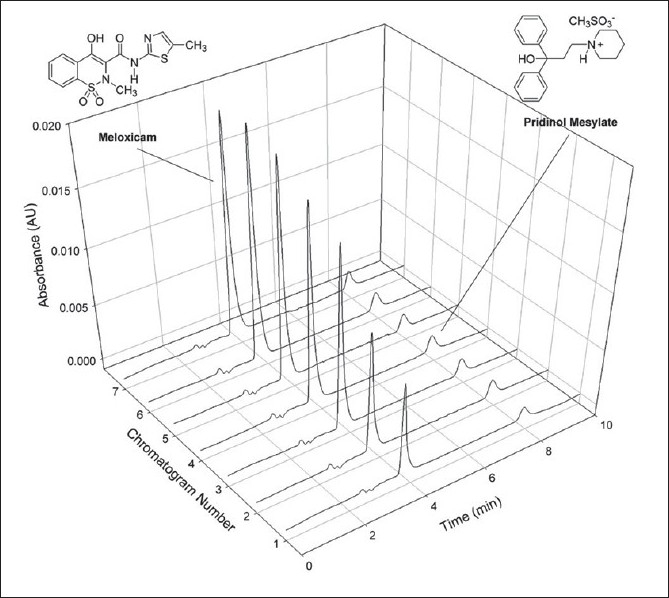
Typical chromatograms of a dissolution curve. Chromatograms of MEL (meloxicam, left peak) and PRI (pridinol, right peak) under the optimized conditions, at different times

**Fig. 6:**
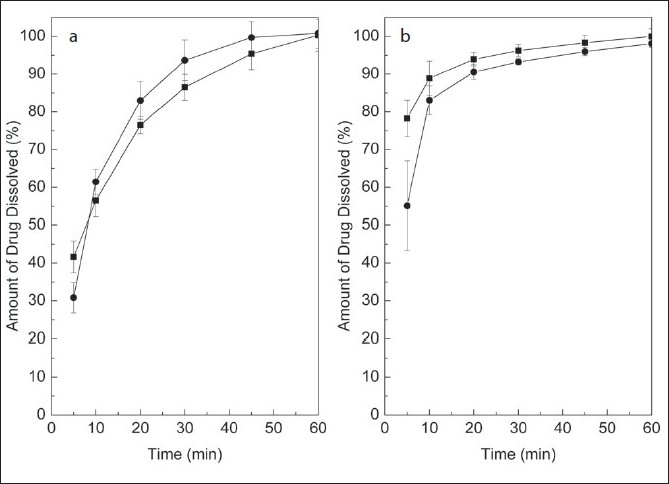
Application of the validated dissolution test to commercial tablets. Dissolution profi les of two brands of tablets containing meloxicam (a) and pridinol (b). Brand 1 (—■—) and Brand 2 (—●—).

The validated dissolution test was applied to the quality control of two commercial brands of tablets containing MEL and PRI, at dissolution profile and single point levels. Fig. 5 depicts typical chromatograms of the dissolution of both drugs at different times, which allowed the construction of the corresponding dissolution curves, while fig. 6 exhibits the dissolution profiles of both drugs in the tested formulations.

For routine work, however, a single point determination of the amount of the dissolved drugs at a pre-specified time is considered satisfactory. According to the BP, a suitable level is Q= 75% of dissolved drug after 45 min[19]. Fig. 6 evidences that under the proposed test conditions, both brands fully complied with this typical specification.

In conclusion, the dissolution stage of a dissolution test for meloxicam and pridinol mesylate in their combined tablet formulation has been developed and validated. The optimum conditions found were 900 ml of 50 mM phosphate buffer (pH= 7.5) as dissolution medium in a bath preheated at 37.0±0.5°, employing USP apparatus 2 with a paddle rotation speed of 75 rpm. Attainment of sink conditions as well as method precision and robustness were demonstrated and the dissolution samples evidenced to be stable for 24 h. Simultaneous analyses of the dissolved drugs were performed employing a suitable HPLC method. Under these validated conditions, two commercial brands of the MEL-PRI association were tested, and their dissolution profiles were also constructed and compared. Both brands demonstrated to release at least 75% of MEL and PRI at the 45 min time point. Because there is no official monograph for dissolution testing of the meloxicam-pridinol combined tablet formulation, the validated dissolution test, which demonstrated to be suitable for its intended purpose, may find use in routine quality control work of this pharmaceutical association.
